# Gastric cancer complicated by paraneoplastic neurological syndrome which presented with extremity numbness: a case report

**DOI:** 10.1186/s40792-022-01429-2

**Published:** 2022-04-28

**Authors:** Takuto Yoshida, Hideki Kawamura, Kazuhiro Mino, Yuji Konishi, Tomoya Saito, Yuichi Shimizu, Akinobu Taketomi

**Affiliations:** 1 Department of General Surgery, Hokkaido Medical Center, 1-1, 5-7 Yamanote, Nishi-ku, Sapporo, 063-0005 Japan; 2 Department of Gastroenterology, Hokkaido Medical Center, 1-1, 5-7 Yamanote, Nishi-ku, Sapporo, 063-0005 Japan; 3 Department of Gastroenterological Surgery 1, Graduate School of Medicine, Hokkaido University, N-15, W-7, Kita-ku, Sapporo, 060-8638 Japan

**Keywords:** Gastric cancer, Paraneoplastic neurological syndrome, Neurological disorder, Numbness

## Abstract

**Background:**

Paraneoplastic neurological syndromes refer to a group of neurological disorders, which occur as distant effects of malignant tumors and are not caused by metastasis, nutritional disorders, or side effects of antitumor drugs.

**Case presentation:**

A 70-year-old woman complained of a 1-month history of extremity numbness. Upon presentation to our hospital, she had worsening numbness, and experienced staggering and falling. Physical examination revealed diminished tendon reflexes in both lower limbs, stocking and glove-type abnormal sensation, and left-sided dominant high-steppage gait due to weakness of the bilateral tibialis anterior muscles. Blood tests indicated anemia, and upper gastrointestinal endoscopy revealed gastric cancer, leading to laparoscopic distal gastrectomy. A nerve conduction velocity test showed demyelinating peripheral neuropathy. Further blood tests and imaging studies ruled out nutritional disorders, such as vitamin deficiency, diabetes-related diseases, connective tissue diseases, and central nervous system metastasis, leading to the suspicion of paraneoplastic neurological syndrome. After laparoscopic distal gastrectomy, the progression of symptoms stopped, and with intravenous high-dose immunoglobulin and steroid therapy, the symptoms improved to only minor numbness in the peripheral limbs as of the 18-month follow-up. As of the 2-year follow-up, there has been no cancer recurrence or metastasis.

**Conclusions:**

When paraneoplastic neurological syndrome is suspected, early diagnosis and a multidisciplinary approach, including surgical treatment, are important before irreversible neurological damage occurs.

## Background

Paraneoplastic neurological syndromes (PNS) refer to a group of neurological disorders, which occur as distant effects of malignant tumors and are not caused by metastasis, nutritional disorders, or side effects of antitumor drugs. Here, we report a case of gastric cancer with PNS diagnosed based on numbness in the extremities. We also review relevant previous reports and summarize case patient findings and outcomes from the literature and the current case in a table (Table 1).

**Table 1:** Description of case studies from the literature

References	Age/sex	Time to diagnosis	Clinical syndrome	Histological type	Onconeural antibody	Neurological outcome	Oncological outcome
Balducci et al. [18]	58/male	1 month	PCD	Neuroendocrine	N/A	Partial response (OP + IT)	Disease free (18 months)
Kikuchi et al. [19]	63/male	N/A	PCD	Neuroendocrine	Ri	N/A	N/A
Baraller et al. [20]	59/male	1 month	OMS	N/A	N/A	No response (IT)	Dead (6 months)
Meglic et al. [21]	73/male	4 months	PCD	Poorly	Yo	No response (OP)	Disease free (6 months)
Wada et al. [22]	64/male	9 months	PLE	Neuroendocrine	Not detected	No response (CT)	Dead (19 months)
Tanaka et al. [23]	77/male	2 months	PLE	N/A	GluR	Progressive (No treatment)	Alive (3 months)
Goto et al.[24]	71/male	-7 months	PCD	N/A	Yo	No response (IT)	Disease free (2 months)
Yasuda et al. [25]	72/female	24 months	SSN, SM	Signet-ring cell	N/A	Complete response (OP + IT)	Disease free (36 months)
Murakami et al. [26]	63/female	5 months	SSN	Neuroendocrine moderate	Hu	Partial response (OP + CT + IT)	Disease free (31 months)
Taketa et al. [3]	72/male	0.5 months	PLE	Neuroendocrine	N-type VGCC	Complete response (OP + CT)	Disease free (15 months)
Biotti et al. [26]	61/male	3 months	PLE	Adenocarcinoma	Ma	Partial response (CT + IT)	Alive (9 months)
Al-Harbi et al. [28]	38/female	2 months	PLE	Neuroendocrine	N/A	Partial response (IT)	N/A
Bataller et al. [29]	59/male	N/A	OMS	Adenocarcinoma	N/A	No response (IT)	Dead (6 months)
Uneno et al. [30]	71/male	N/A	PLE	Adenocarcinoma	Hu	Partial response (CT)	Dead (14 months)
Our case	70/female	1 months	LEMS	Adenocarcinoma	N/A	Complete response (OP + IT)	Disease free (24 months)

*N/A* not applicable; *PCD* paraneoplastic cerebellar degeneration; *OMS* opsoclonus–myoclonus; *PLE* paraneoplastic limbic encephalitis; *SSN* subacute sensory neuropathy; *SM* systematic myositis; *VGCC* voltage-gated calcium channel; *LEMS* Lambert–Eaton myasthenic syndrome; *OP* operation; *IT* immunotherapy; *CT* chemotherapy; *GluR* glutamate receptor

## Case presentation

**Fig. 1:**
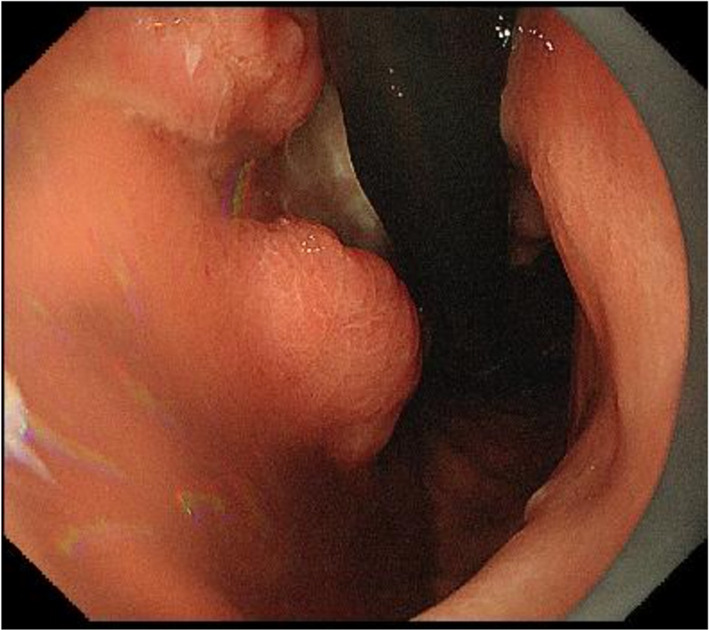
Gastric fiber. Type 3 tumor in posterior wall of the lower part of the stomach

**Fig. 2:**
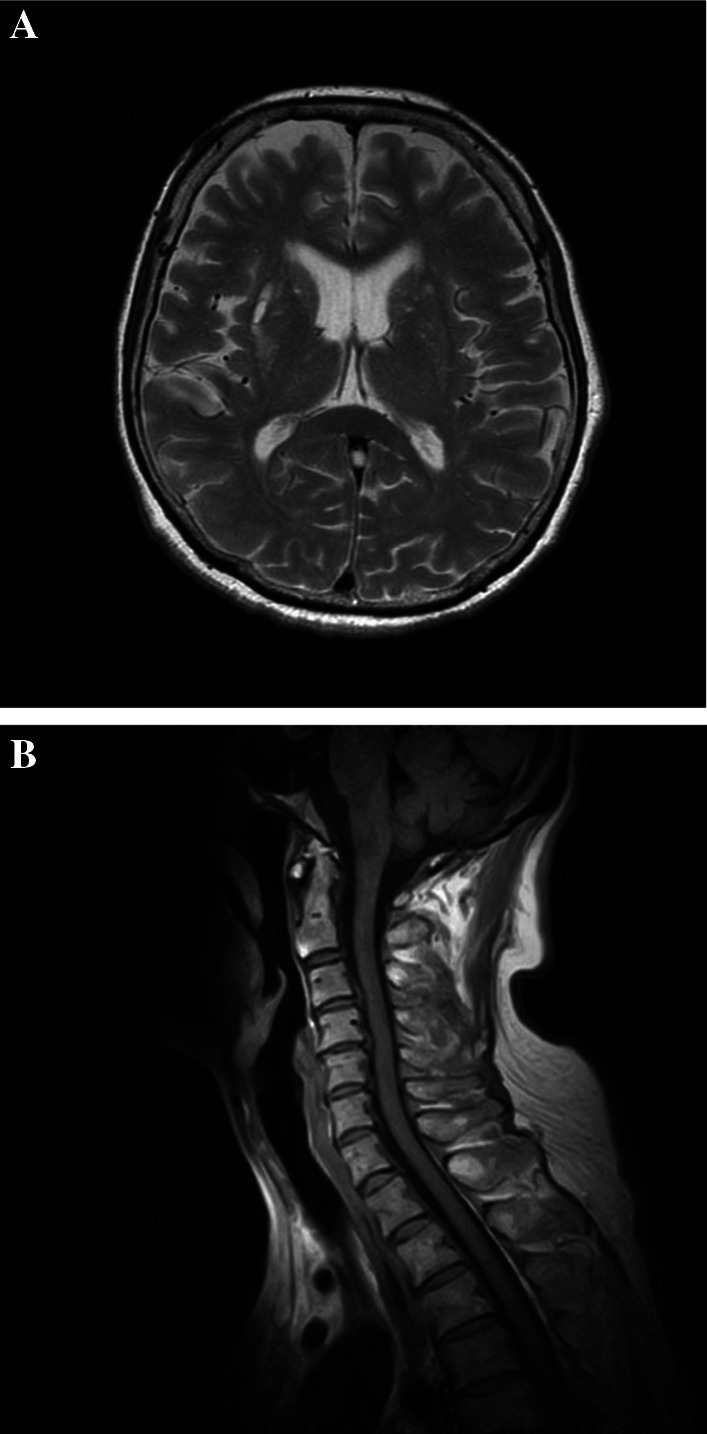
Magnetic resonance imaging. **A** Brain magnetic resonance imaging (MRI). Brain MRI shows multiple T2 high signals in the bilateral basal ganglia and cerebral white matter, indicating a chronic ischemic lesion. There is no evidence of brain metastasis. **B** Spinal MRI. C4 kyphosis and narrowing of the spinal canal of C4/5. No obvious spinal cord compression or abnormal signal is observed

**Fig. 3:**
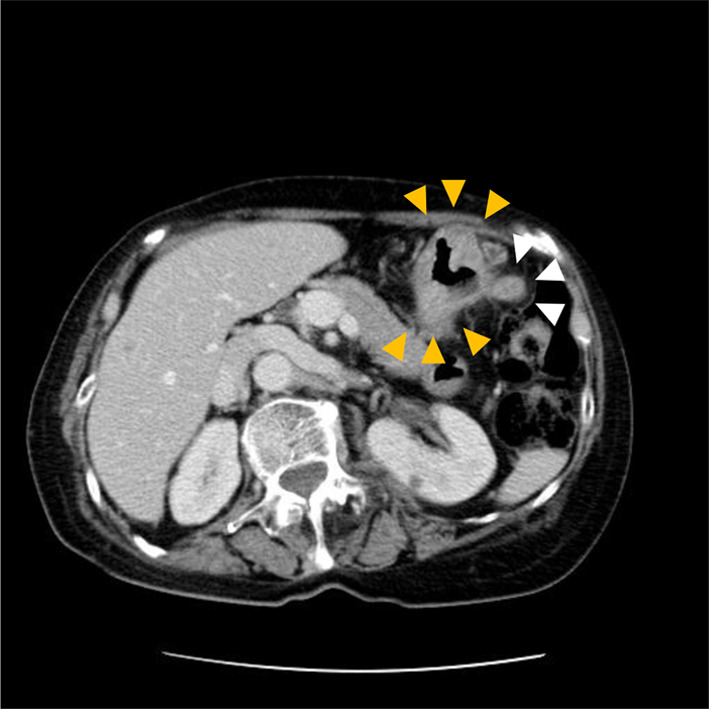
Abdominal CT. There is irregular wall thickening with a contrast effect on the posterior wall of the lower stomach (yellow arrow). Multiple enlarged lymph nodes are observed on the greater curvature of the stomach (white arrow). No obvious distant metastasis is observed

**Fig. 4:**
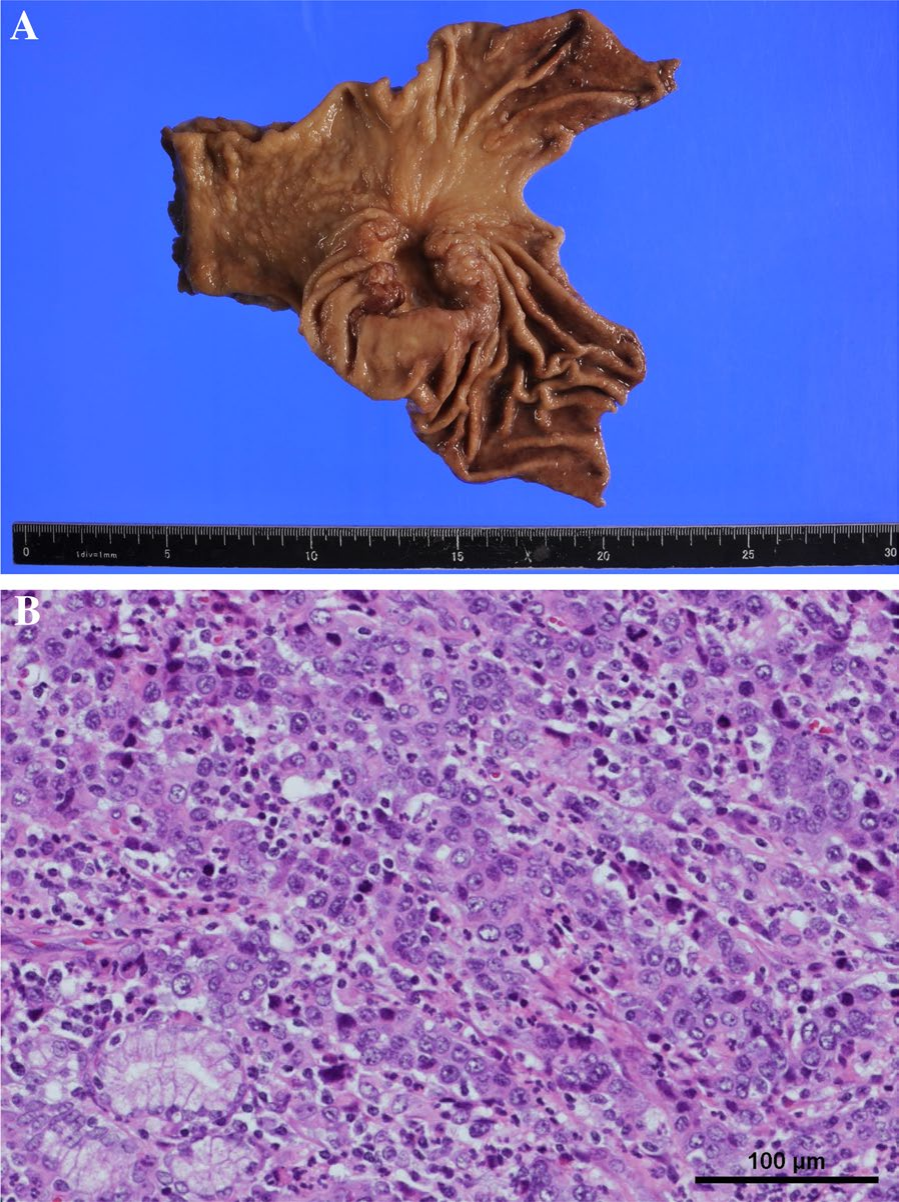
Pathophysiology. **A** Pathological specimen. A 45 × 40 mm type 3 lesion was found 2.5 cm from the proximal margin and 7 cm from the distal margin. **B** Hematoxylin–Eosin double stain, × 20. Histologically, the lesion shows moderately to poorly differentiated adenocarcinoma

A 70-year-old woman presented with a 1-month history of bilateral lower extremity numbness with left-sided predominance. She had a history of hypertension and insomnia, and was taking candesartan and triazolam. Blood tests showed a hemoglobin (Hb) level of 7.8 mg/dl, and an upper gastrointestinal endoscopy revealed a type 3 lesion on the posterior wall of the lower part of the stomach, which was biopsied and diagnosed as adenocarcinoma (Fig. 1). At that time, numbness had appeared in both hands. All symptoms gradually worsened, resulting in staggering, falling, and difficulty in walking. Physical examination revealed diminished tendon reflexes in the lower extremities and decreased muscle strength (manual muscle test [MMT] 3/5) of the bilateral tibialis anterior muscles. The Romberg sign was fair, and a bilateral steppage gait was observed. Blood test results were: vitamin B12, 197; antinuclear antibody (ANA) titer, < 40; anti-deoxyribonucleic acid (DNA) antibody, < 2.0; anti-SS-A antibody, < 1.0; anti-SS-B antibody, < 1.0; soluble IL-2 receptor, 321; myeloperoxidase–anti-neutrophil cytoplasmic antibodies (MPO–ANCA), < 1.0; and proteinase 3–anti-neutrophil cytoplasmic antibodies (PR3–ANCA), < 1.0. Cerebrospinal fluid (CSF) examination revealed glucose of 58 mg/dl, cell count (1/3, mononuclear cells 100%), protein 122 mg/dl, and Cl 125 mEq/l. PNS and chronic inflammatory demyelinating polyneuropathy were considered as differential diagnoses. Brain magnetic resonance imaging (MRI) did not reveal any findings suggestive of brain metastasis (Fig. 2A). Spinal MRI showed kyphotic deformity of C4 and narrowing of the spinal canal around C4/5, but no obvious spinal cord compression or abnormal intramedullary signals (Fig. 2B). Contrast-enhanced computed tomography (CT) of the abdomen revealed wall thickening with contrast effect on the lateral side of the greater curvature of the stomach and enlarged lymph nodes in the surrounding area, suggesting metastasis (Fig. 3). Nerve conduction velocity testing showed decreased amplitude of complex-type action potentials (CMAP) in the ulnar and peroneal nerves and no derivation of F waves. A laparoscopic distal gastrectomy was performed. The pathological results showed a 45 × 40 mm type 3 lesion on the posterior wall of the mid-gastric body. Histologically, the lesion appeared to be a moderately to poorly differentiated adenocarcinoma, with tumor cells infiltrating the subserosal layer and invading lymphatic vessels and veins (Fig. 4). Based on the pathological examination, the final diagnosis was T3N1M0 Stage IIB, according to the Japanese Classification of Gastric Carcinoma (JCGC) (3^rd^ English Edition) [1]. The postoperative course was uneventful; intravenous high-dose immunoglobulin therapy (IVIG) was started on the third postoperative day, and oral steroids were started on the 14th postoperative day. After rehabilitation, the patient was able to walk with a walker and the numbness in the limbs disappeared, and she was discharged on the 28th day after surgery. Her advanced age and prolonged hospitalization resulted in a marked decline in her physical activity, and we determined that adjuvant chemotherapy was not indicated according to her request. Steroids were then started at 25 mg but reduced by 5 mg every 4 weeks and continued at 10 mg for some time. At 8 months postoperatively, we decreased the dose to 5 mg, and after that, we reduced the steroid volume by 1 mg every 4 weeks, and the steroids were terminated at 18 months postoperatively. With this dosage regimen, the patient was able to walk with a cane 1 month after discharge from the hospital, and another month after that, he no longer needed a cane. The decreased deep tendon reflexes and mild steppage gait remained for some time but completely disappeared at 18 months postoperatively. Two years have passed since the surgery without any recurrence or metastasis.

## Discussion

PNS are seen in patients with malignant tumors and are exclusive of tumor invasion and metastasis, nutritional and metabolic disturbances, and side effects of antitumor drugs. PNS are stated to be “remote effects of cancer on the nervous system” [2]. Broadly, PNS refer to the remote effects of malignancy on organs and tissues other than the primary tumor site. However, in a narrower sense, the syndromes do not include metabolic conditions, such as hypercalcemia associated with malignancy. Rather, only immune-mediated neurological disorders are included. Among patients with malignant tumors, PNS are more common in those with small cell carcinoma of the lung; the incidence in other solid tumors is much lower than 1%, and it is considered a rare condition [3]. PNS can present with the following neurological manifestations: encephalomyelitis, limbic encephalitis, paraneoplastic cerebellar degeneration, opsoclonus/myoclonus, sensory neuropathy, chronic intestinal pseudo-obstruction, and Lambert–Eaton myasthenic syndrome (LEMS), and are called "classical syndromes" which have a robust association with tumors [4, 5]. In the current case, the patient had muscle weakness and decreased deep tendon reflexes in the lower extremities, suggesting Lambert–Eaton myasthenic syndrome. Lambert–Eaton myasthenic syndrome is often associated with small cell carcinoma of the lung but is also seen in patients with gastric cancer and thymoma [6]. Antibodies against P/Q-type voltage-gated calcium channels (VGCCs) and other voltage-gated calcium channels are common [7]; however, antibodies against synaptotagmin [8], amphiphysin [9], and CRMP-5 [3] have also been reported. These antibodies were not detected in this case.

Approximately two-thirds of patients suspected of having PNS have neurological symptoms, but no diagnosis of cancer [10]. In a review of 10 patients with PNS, Taketa et al. reported that, on average, neurological symptoms were present approximately 6 months before the diagnosis of cancer [11]. If PNS is suspected in such patients, it is necessary to measure PNS-related antibodies and search for cancer. Although the presence of PNS-related antibodies is highly suggestive of the presence of tumors, the positivity rate is less than 1% [12, 13]. Therefore, even if the test is negative, the patient should be retested at 3 and 6 months. In addition, the European Federation of Neurological Societies (EFNS) Task Force recommends periodic searches every 6 months for 4 years [14]. When a patient diagnosed with cancer develops neurological symptoms, the diagnosis of PNS is made after ruling out metastasis to the central nervous system via imaging and CSF studies. There are three main groups of PNS-associated antibodies: "well-characterized onconeural antibodies," such as anti-Hu and anti-Yo antibodies, in which neurological symptoms are strongly considered paraneoplastic and are strongly associated with certain cancers; a group that is not necessarily associated with the presence of cancer; and a group known as "partially-characterized onconeural antibodies," in which significance as markers of PNS has been confirmed, although the characteristics of the corresponding antigen are not precise [4]. In the current case, neurological symptoms preceded the disease. However, anemia was also observed in blood tests, which led to upper gastrointestinal endoscopy and early diagnosis. Although specific antibodies were negative, metastasis to the central nervous system was ruled out, leading to an early diagnosis of PNS.

The EFNS Task Force considers the search for and treatment of malignancy, immunosuppression, and symptom control as the mainstays of therapy [14]. Resection of malignant tumors is the most effective treatment to control the progression of neurological symptoms and, in particular, to restore function [10, 15, 16]. Immunosuppressive treatment includes plasma exchange and large doses of immunoglobulins, corticosteroids, rituximab, and tacrolimus [17]. PNS often lead to irreversible neurological damage by the time of therapeutic intervention, and the treatment prognosis is generally poor. In several reports, PNS have been caused by gastric cancer, including our case (Table 1). Taketa et al. [10] reported the following characteristics of gastric cancer associated with PNS: first, approximately half of the cases are neuroendocrine tumors (NET) or undifferentiated carcinomas, such as signet ring cell carcinoma derived from neuroendocrine tumors. In the present case, postoperative pathological examination revealed the presence of poorly differentiated adenocarcinoma, which is consistent with previous reports. Second, although NET is usually considered to have a poor prognosis, when accompanied by PNS, the prognosis is relatively good, with an excellent response to treatment. This is due to the expression of major histocompatibility complex (MHC) class 1 and non-amplification of the myc gene. To date, the current case is under observation without recurrence. The third point is that it takes an average time of 5.8 months from the onset of symptoms to diagnosis. However, in the present case, gastric cancer was diagnosed within approximately 1 month from the onset of symptoms, and we were able to intervene early with treatment. In addition to surgical intervention, there are many reports of patients being treated with chemotherapy and immunosuppressive agents that have shown therapeutic efficacy. However, although there are few case reports of complete response, in the current case, early diagnosis and resection of the primary lesion, followed by prompt introduction of immunosuppressive agents, may have contributed to the disappearance of neurological symptoms. Conversely, considering approximately 2 years of oral immunosuppressive medication was necessary to achieve complete resolution of symptoms, the importance of early diagnosis is apparent. When PNS is suspected, early diagnosis, early surgery, and immunosuppressive therapy are considered necessary.

## Conclusions

We experienced a patient with gastric cancer complicated by rare PNS. When PNS is suspected, early diagnosis and treatment are important before irreversible neurological damage occurs.

## Data Availability

The data set supporting the conclusions of this article is included in the article.

## Abbreviations

PNS: Paraneoplastic neurological syndrome
MMT: Manual muscle test
ANA: Antinuclear antibody
DNA: Deoxyribonucleic acid
MPO–ANCA: Myeloperoxidase–anti-neutrophil cytoplasmic antibodies
PR3–ANCA: Proteinase 3–anti-neutrophil cytoplasmic antibodies
MRI: Magnetic resonance imaging
CSF: Cerebrospinal fluid
CT: Computed tomography
CMAP: Complex-type action potentials
IVIG: Intravenous high-dose immunoglobulin therapy
LEMS: Lambert–Eaton myasthenic syndrome
VGCC: Voltage-gated calcium channel
NET: Neuroendocrine tumors
EFNS: The European Federation of Neurological Societies
MHC: Major histocompatibility complex
